# Evaluation of the Protective Effect of n-Hexane Oil Extract of Black Soldier Fly Larvae on Oxidative Stress, Inflammation, and Signaling Pathway Alterations in Acrylamide-Induced Hepatotoxicity in Rats

**DOI:** 10.5812/ijpr-171556

**Published:** 2026-06-17

**Authors:** Yalda Arast, Arash Parvaresh, Somaye Behnamipour, Fateme Heidari, Tahereh Zarei Taher, Jalal Pourahmad

**Affiliations:** 1 Research Center of Environmental Pollutants, Qom University of Medical Sciences, Qom, Iran; 2 Department of Pharmacology and Toxicology, School of Pharmacy, Shahid Beheshti University of Medical Sciences Tehran, Tehran, Iran; 3 Department of Tissue Engineering and Applied Cell Sciences, Student Research Committee, School of Advanced Technologies in Medicine, Shahid Beheshti University of Medical Sciences, Tehran, Iran; 4 Department of Anatomical Sciences, School of Medicine, Qom University of Medical Sciences, Qom, Iran; 5 Department of Anatomical Sciences, Faculty of Medicine, Ramsar Campus, Mazandaran University of Medical Sciences, Ramsar, Iran; 6 Department of Tissue Engineering and Applied Cell Sciences, Faculty of Medicine, Qom University of Medical Sciences, Qom, Iran; 7 Student Research Committee, Qom University of Medical Sciences, Qom, Iran; 8 Department of Pharmacology and Toxicology, School of Pharmacy, Shahid Beheshti University of Medical Sciences, Tehran, Iran

**Keywords:** Acrylamide, *Hermetia illucens*, Oxidative Stress, Apoptosis, Liver Injury, GRP78-mediated Endoplasmic Reticulum Stress-signaling Pathway

## Abstract

**Background:**

The liver, the body’s central organ for metabolism and detoxification, is continually exposed to environmental and dietary toxins such as acrylamide (ACR). ACR is commonly found in roasted and fried foods. Strong scientific evidence indicates that ACR exposure causes serious liver injury. Black soldier fly larvae (BSFL) are an environmentally friendly insect, and their extract is rich in valuable bioactive compounds with antioxidant properties.

**Objectives:**

This study aimed to investigate the protective effects of the n-hexane extract of black soldier fly larvae against oxidative stress, inflammation, and histopathological changes associated with ACR-induced hepatotoxicity in rats.

**Methods:**

Thirty-five male Wistar rats weighing 200 to 250 g were randomly assigned to five equal groups: control, ACR (20 mg/kg), BSFL (360 mg/kg), BSFL (180 mg/kg) + ACR, and BSFL (360 mg/kg) + ACR. At the end of treatment on the twenty-eighth day, the animals were euthanized, and samples were collected for assessment of liver enzymes, oxidative stress, inflammation, ER stress, apoptosis markers, histopathology, and endoplasmic reticulum stress-related protein expression. Data were analyzed using one-way ANOVA followed by Tukey's post-hoc test, with P < 0.05 considered statistically significant.

**Results:**

ACR administration significantly increased AST, ALT, ALP, MDA, and NO levels (P < 0.001), significantly decreased SOD, GSH, GPx, and CAT levels (P < 0.001), and significantly increased TNF-α, IL-1β, and IL-6 levels (P < 0.001) compared with the control group. These ACR-induced biochemical and inflammatory abnormalities were confirmed by histopathological observations of liver tissue. Administration of BSFL extract, at the more potent dose of 360 mg/kg, significantly reversed these biochemical, inflammatory, and hepatic markers. BSFL extract was associated with changes in ER stress-related proteins, apoptosis markers, NF-κB, and MAPK family members, suggesting a potential role in GRP78-mediated ER stress signaling. Histological results showed that BSFL extract (360 mg/kg) reduced steatosis, cellular swelling, and lobular inflammation induced by ACR exposure.

**Conclusions:**

The results of this study indicate that BSFL extract, a valuable bioactive substance with antioxidant properties, may attenuate alterations in biochemical indices and reduce oxidative stress and inflammation caused by ACR-induced hepatotoxicity.

## 1. Background

The liver, the body’s central organ for metabolism and detoxification, is continuously exposed to environmental and dietary toxins such as acrylamide (ACR). ACR is commonly present in roasted and fried foods. Strong scientific evidence indicates that ACR exposure causes serious liver injury, rendering the liver highly vulnerable to chemical damage. ACR is a major global concern because it is widely used in industry and is also formed unintentionally in carbohydrate-rich foods (e.g., fries or baked goods) cooked at high temperatures (1, 2). Strong scientific evidence shows that ACR exposure causes serious liver injury through a damaging chain reaction:

ACR-induced damage includes severe oxidative stress: ACR triggers the generation of reactive oxygen species and depletes endogenous hepatic antioxidants (e.g., glutathione, superoxide dismutase, and catalase) (3).

ACR-induced damage includes harmful inflammation: It disrupts signaling pathways (especially NF-κB), leading to increased inflammatory mediators (e.g., TNF-α and IL-6) (4).

ACR-induced damage includes physical liver damage: This oxidative stress and inflammation cause overt tissue injury, including cell death, fat accumulation, and hepatic inflammation (5).

Although the WHO sets a guideline limit for ACR in drinking water (0.5 mg/L), no such limits exist for ACR in food (6). Research by SNFA and Stockholm University supports this concern, reporting medium levels (5 - 50 mg/kg) in heated proteins and high levels (150 - 4000 mg/kg) in cooked carbohydrate-rich foods, whereas boiled or uncooked foods contain no ACR (7).

This clear link between ACR and liver damage—via oxidative stress, inflammation, and tissue breakdown—highlights the urgent need for protective strategies. Concurrently, there is growing interest in sustainable natural compounds with therapeutic potential. One promising yet understudied source is insects, specifically black soldier fly larvae (BSFL) (8).

BSFL oil is rich in bioactive lipids, including lauric acid, other beneficial fatty acids, tocopherols (vitamin E), sterols, and phospholipids (9). In vitro tests and early animal studies suggest that this oil has potent antioxidant and anti-inflammatory effects. Oil extracted with n-hexane concentrates these beneficial components, potentially enabling it to target key pathways involved in chemical liver injury, such as enhancing endogenous antioxidant defenses (via nuclear factor erythroid 2-related factor 2, Nrf2) and attenuating inflammation (by suppressing NF-κB) (10, 11).

The selection of BSFL oil for this investigation was based on several advantages over other natural sources. First, BSFL are highly efficient bioconverters, converting organic waste into biomass rich in high-value lipids. This provides a sustainable and scalable source that does not compete with the human food chain, unlike many plant-derived extracts. Second, the lipid profile of BSFL oil is distinctive, with a high content of medium-chain fatty acids (MCFAs) such as lauric acid. Lauric acid and its metabolite, monolaurin, are well documented for their potent antioxidant and anti-inflammatory activities. Moreover, the n-hexane fraction specifically concentrates these non-polar bioactive compounds, potentially offering more targeted efficacy. Therefore, BSFL extract is not merely an alternative; it is a promising, sustainable, and potent candidate warranting specific investigation for hepatoprotection.

## 2. Objectives

Therefore, to address this critical gap, the present study was designed using a novel, multi-mechanistic approach. We hypothesized that the BSFL extract would confer hepatoprotection against ACR by simultaneously targeting the interconnected pathways of oxidative stress, inflammation, and cellular apoptosis. As an essential first step, we deliberately used the total n-hexane extract to assess the cumulative and potentially synergistic hepatoprotective effects of its complex mixture of bioactive lipids, which is particularly rich in medium-chain fatty acids, such as lauric acid (constituting up to ~50% of its fatty acid profile), and also contains antioxidant components, including tocopherols (9, 10). For the first time, we aimed to investigate not only the biochemical and histological outcomes but also to elucidate the underlying molecular mechanism by focusing on the endoplasmic reticulum stress-mediated signaling pathway (GRP78/ATF6/IRE1), a key driver of ACR-induced hepatotoxicity. By evaluating the effects of BSFL extract across this comprehensive set of parameters, our study provides foundational evidence supporting the potential of this sustainable insect-derived extract as a multifaceted therapeutic agent against dietary and environmental toxicant-induced liver injury.

## 3. Methods

### 3.1. Ethical Approval

This study was approved by the Research Ethics Committee of Qom University of Medical Sciences (Approval Code: IR.MUQ.AEC.1404.012) and Shahid Beheshti University of Medical Sciences (Approval Code: IR.SBMU.PHARMACY.REC.1403.156). All procedures were conducted in accordance with institutional guidelines for animal welfare.

### 3.2. Animals and Housing

Thirty-five male Wistar rats (200 - 250 g) were obtained from the Animal Studies Center, Qom University of Medical Sciences. Animals were housed under controlled conditions (21 ± 2°C, 12-h light/dark cycle) with free access to standard pellet feed and water. Cages were cleaned daily. Rats were housed individually to prevent cross-interaction and to enable individual oral gavage administration. Each animal was identified by ear marking.

### 3.3. Materials

Black Soldier Fly Larvae (BSFL) Oil Extract: The n-hexane extract of Hermetia illucens larvae was provided under the approved technological proposal of Qom University of Medical Sciences. The n-hexane extract was prepared using the Soxhlet extraction method. The resulting oil was concentrated under reduced pressure using a rotary evaporator, and the solvent was completely removed. The final extract was stored at -20°C until use. The n-hexane solvent was specifically selected for its high affinity and efficiency in extracting non-polar lipid components. This method is particularly effective for obtaining a high yield of the oil fraction rich in medium-chain fatty acids, such as lauric acid, which is documented as the predominant bioactive compound in BSFL oil, constituting a substantial portion of its fatty acid profile (10). Therefore, n-hexane was selected to maximize the extraction of these lipid-soluble bioactive compounds hypothesized to mediate hepatoprotective effects.

Chemicals: Acrylamide (Sigma-Aldrich, USA), corn oil (vehicle), formaldehyde, and all assay kits were commercially sourced. Acrylamide and BSFL extract were dissolved in corn oil prior to administration.

### 3.4. Experimental Design

Rats were randomly assigned to experimental groups using a random number table to minimize bias. The random allocation sequence was generated by a researcher not involved in animal handling or data collection. Allocation was concealed using sequentially numbered, opaque, sealed envelopes. On the first day of treatment, a technician who was not involved in outcome assessment opened the envelopes and assigned animals to their respective groups. After one week of acclimatization, rats were randomly divided into five groups (n = 7/group). Sample size justification: This was a preliminary proof-of-concept study. An a priori power analysis was not performed. The sample size of n = 7 per group was selected based on (1) consistency with minimum recommendations for pilot hepatotoxicity studies, (2) comparability with previous similar studies (n = 6), and (3) post-hoc sensitivity analysis (G*Power, effect size f=0.6, α=0.05, power=0.80) confirming adequacy for detecting large effect sizes.

Control (Corn Oil): Received 1 mL/kg/day corn oil orally.

ACR Group: Received 20 mg/kg/day ACR orally (12).

BSFL Group: Received 360 mg/kg/day BSFL extract orally alone (13).

ACR + BSFL 180 Group: Received 20 mg/kg/day ACR + 180 mg/kg/day BSFL extract orally (BSFL administered first, followed within minutes by ACR).

ACR + BSFL 360 Group: Received 20 mg/kg/day ACR + 360 mg/kg/day BSFL extract orally (BSFL administered first, followed within minutes by ACR).

The selection of BSFL extract doses (180 and 360 mg/kg) was based on a translational rationale informed by the known bioactive composition of the extract and existing pharmacological data. Given that lauric acid constitutes the predominant fatty acid (≥70%) in BSFL oil (5), the dosing strategy was designed to deliver a therapeutically relevant amount of this key component. The chosen doses correspond to an estimated daily intake of approximately 100 mg/kg and 200 mg/kg of lauric acid, respectively. This range is consistent with the effective dosage range of lauric acid (100 - 250 mg/kg) previously demonstrated to exert significant hepatoprotective effects in rodent models of toxin-induced liver injury (13). Furthermore, the higher dose (360 mg/kg) was included to evaluate a potential dose-response relationship and to ensure a robust test of the extract's efficacy.

Treatments were administered daily for 28 days. Body weights were recorded at baseline and on day 29.

Blinding statement: Owing to limited personnel and resource constraints in this preliminary study, complete blinding was not achieved. The investigator performing oral gavage was aware of group allocations because different treatment volumes were prepared for each group. However, the technician conducting biochemical assays and the investigators performing western blot densitometry were blinded to group allocation using coded sample labels. Histopathological assessment was performed by a pathologist who was not blinded due to the overt nature of ACR-induced histopathological changes; nevertheless, predefined semi-quantitative scoring criteria were applied to minimize subjective bias. This limitation is acknowledged in the Discussion section.

### 3.5. Sample Collection

On day 29, rats were anesthetized with ketamine (50 mg/kg) and xylazine (10 mg/kg). Blood was collected via cardiac puncture into sterile tubes. Serum was separated by centrifugation (4000 × g, 10 min, 4°C) and stored at -20°C for liver function analysis. Liver tissues were rapidly excised. Sections were fixed in 10% neutral buffered formalin for ≥ 24 h for histopathology. The remaining tissue was homogenized (1:10 w/v) in ice-cold [phosphate buffer saline or Tris-HCl], centrifuged (12,000 × g, 15 min, 4°C), and the supernatant was stored at -80°C for biochemical assays. Protein concentration was determined using the Bradford method (Bradford, 1976) (12).

### 3.6. Biochemical Assays

Liver Function Markers (Serum): ALT, AST, and ALP levels were measured using commercial colorimetric kits (Wiesbaden, Germany) and a spectrophotometer (UNICOInstruments C., Model 1200, USA).

### 3.6.1. Oxidative Stress Markers (Liver Homogenate)

Lipid peroxidation: Malondialdehyde (MDA) levels were evaluated using the MDA assay kit according to the manufacturer's protocol (Teb Pazhouhan Razi (TPR), Tehran, Iran). Liver NO levels were measured using the Griess diazotization reaction after conversion of nitrate to nitrite by nitrate reductase in the supernatant (Nitric Oxide Assay Kit, Navand Lab Kit, Tehran, Iran).

Antioxidant enzymes: Catalase (CAT) activity was measured using a kit (Catalase Activity Assay Kit, Navand Lab Kit, Tehran, Iran). *Superoxide dismutase* (SOD) activity was evaluated using the SOD assay kit according to the manufacturer's protocol (*Superoxide dismutase* (SOD) Activity Assay Kit, Navand Lab Kit, Tehran, Iran).

The method described by Ellman (Ellman 1959) was used for glutathione S-transferase (GST) analysis (GSH Activity Assay Kit, Navand Lab Kit, Tehran, Iran). *Glutathione peroxidase* (GPx) activity was measured using the GSH peroxidase kit (GPx Activity Assay Kit, Navand Lab Kit, Tehran, Iran).

Inflammatory Markers (Liver Homogenate): Levels of hepatic inflammatory factors, including TNF-α, IL-1β, and IL-6, were determined by ELISA using the relevant kits (CN: KPG-TNF-α -48, CN: KPG-RIL1β, CN: KPG-RIL6, Karmania Pars Gene, Tehran, Iran) and IL-1b Assay Kit (IBL Company, Code No. 27193), and the results were expressed as pg/mg of protein.

### 3.7. Western Blot Assay

Western blot analyses were performed as previously described with some modifications (14, 15). For western blotting, tissue was lysed in RIPA buffer. Lysates were cleared by centrifugation at 14,000 rpm for 20 min at 4°C. Protein concentration was determined using the Bradford Protein Quantification kit (DB0017, DNAbioTech, Iran) according to the manufacturer's instructions. Tissue lysates were mixed with an equal volume of 2× Laemmli sample buffer. Lysates (20 µg) were then subjected to SDS-PAGE after boiling for 5 min and subsequently transferred to a 0.2 µm Immune-Blot™ polyvinylidene difluoride (PVDF) membrane (Cat No: 162 - 017777; Bio-Rad Laboratories, CA, USA). Membranes were then blocked with 5% BSA (Cat No: A-7888; Sigma Aldrich, MO, USA) in 0.1% Tween 20 for 1 h.

Membranes were incubated overnight at 4°C with primary antibodies against ASK1 (1:1000), phospho-JNK (1:1000), p38 MAPK (1:1000), GRP78/BiP (1:2500, Cat No: ab8227, Abcam), NF-κB p65 (1:1000), Caspase 12 (1:800), ERK1 (1:1000), and β-actin (1:2500, loading control). Subsequently, membranes were washed three times with TBST and incubated with goat anti-rabbit IgG H&L (HRP) secondary antibody (1/10000, Cat No: ab6721; Abcam) for 1 h at room temperature. Membranes were then developed using enhanced chemiluminescence (ECL) for 1 - 5 min. The following proteins were measured: ASK1, phospho-JNK, p38 MAPK, GRP78/BiP, NF-κB p65, Caspase 12, ERK1, and β-actin (loading control). Protein expression was normalized to β-actin. Densitometry of protein bands was performed using Gel Analyzer software (Version 2010a, NIH, USA), such that the percentage area under the curve of each band was divided by the percentage area under the curve of its corresponding actin band, and the calculated values were then compared between groups as described previously (16).

### 3.8. Histopathological Examination

Liver tissues from experimental rats were harvested and processed for histological examination to assess the protective effects of the n-hexane oil extract from Black Soldier Fly Larvae (BSFL) against acrylamide-induced hepatotoxicity. Five experimental groups were examined: Control, ACR, BSFL 360, ACR + BSFL 180, and ACR + BSFL 360. Sections were stained with Masson's trichrome (for collagen/fibrosis assessment) and Hematoxylin & Eosin (H&E) to evaluate general hepatic architecture and inflammatory cells. Slides were examined at 100× magnification, with central vein (CV) and portal vein (PV) markers for orientation. Histological alterations, including fibrosis, inflammatory infiltration, and hepatocellular integrity, were semi-quantitatively assessed (17).

Scoring was performed by a pathologist as follows: ballooning degeneration (0: none, 1: mild, 2: moderate, 3: severe), lobular inflammation (0: none, 1: mild, 2: moderate, 3: severe), and collagen deposition (0: none, 1: mild, 2: moderate, 3: severe). For each animal, three non-consecutive sections were examined, and ten random fields per section were evaluated at 100× and 400× magnification.

### 3.9. Statistical Analysis

Data are expressed as mean ± SD. Before one-way ANOVA, normality (Shapiro-Wilk, P > 0.05), homogeneity of variances (Levene's test, P > 0.05), and independence (individual housing) were confirmed. No outliers were detected (Grubbs' test, α = 0.05). No missing data or failed assays occurred. All endpoints (biochemical, oxidative stress, cytokine, western blot, histopathological) were pre-specified in the ethics-approved protocol. Differences between groups were analyzed using one-way ANOVA followed by Tukey's post-hoc test in GraphPad Prism. P < 0.05 was considered significant. Given the multiple outcomes tested, P values were interpreted with caution. Tukey's post-hoc test inherently adjusts for multiple comparisons within each family of outcomes (e.g., liver enzymes, oxidative stress markers). No additional adjustment (e.g., Bonferroni) was applied across different outcome families to avoid an excessive risk of type II error, as is common in exploratory preclinical studies. All reported P values are two-tailed. Exact P values are provided in the figure legends for all main comparisons.

## 4. Results

The primary outcomes of this study were serum liver enzymes (ALT, AST, ALP) and hepatic oxidative stress markers (MDA, GSH, SOD, CAT, GPx). All other outcomes (inflammatory cytokines, western blot proteins, histopathological scores) were secondary/exploratory.

### 4.1. Liver Function Markers (Serum)

Figure 1A-C shows biomarkers of hepatic function in rats treated with ACR or BSFL extract alone and in combination. Serum ALT activity increased (P < 0.001) following ACR administration alone compared with the control group. This increase, indicative of hepatotoxicity, was significantly reduced in rats co-treated with ACR and BSFL at 180 and 360 mg/kg (P < 0.05) (Figure 1A). Serum AST levels also increased significantly with ACR treatment (P < 0.001), whereas BSFL at 360 mg/kg decreased AST activity (P < 0.001). No significant difference in AST levels was observed in the group that received BSFL at 180 mg/kg in combination with ACR compared with the ACR group (Figure 1B).

**Figure 1.**
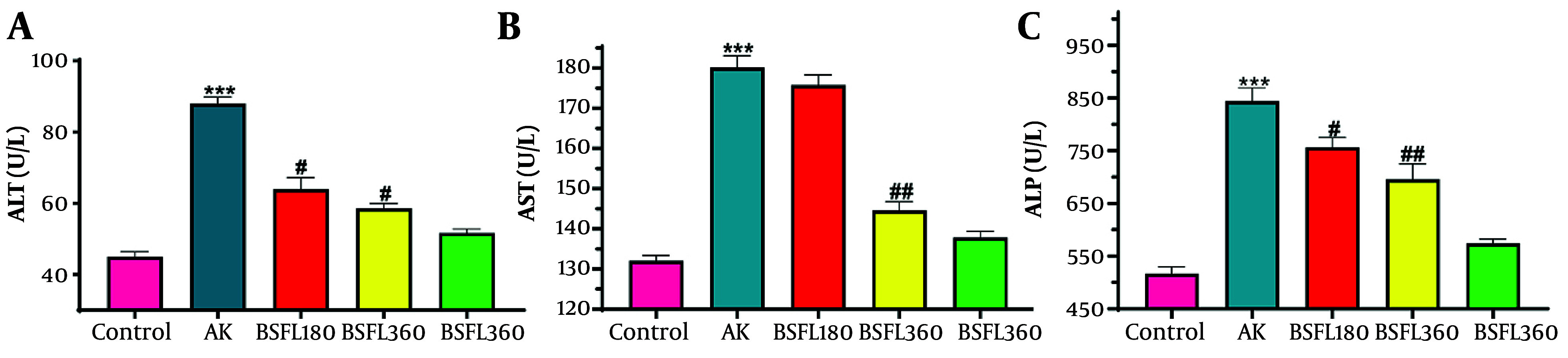
Treatment with n-hexane oil extract of Black Soldier Fly Larvae (BSFL) at two different concentrations (180 and 360 mg/kg) on ALT, AST, and ALP levels in the ACR-induced model. Values are expressed as mean ± SD; n = 7. *Significant difference in comparison with the control group (*** P < 0.001). #Significant difference in comparison with the ACR group (# P < 0.05; ## P < 0.01).

Serum ALP levels also increased significantly after administration of ACR alone, showing a pattern similar to that of ALT and AST, compared with the control group (P < 0.001). The ACR-induced increase in ALP was significantly reduced after treatment with BSFL at 180 and 360 mg/kg (P < 0.05 and P < 0.01). No significant difference in ALT, AST, or ALP levels was observed in the group that received BSFL at 360 mg/kg alone compared with the control group.

### 4.2. Antioxidant Enzymes

#### 4.2.1. Catalase Activity Assay

The effect of the n-hexane oil extract of Black Soldier Fly larvae (BSFL) on hepatic catalase activity (17) in ACR-exposed rats is shown in Figure 2. ACR intoxication severely compromised the antioxidant defense system, as evidenced by a significant decrease in CAT activity. The ACR-only group exhibited a marked reduction in CAT activity to 13.81 nmol/min/mL, compared with 41.88 nmol/min/mL in the normal control group, representing a 67% impairment and confirming severe oxidative stress. Co-treatment with the BSFL extract attenuated this ACR-induced deficit in a dose-dependent manner. The lower dose (180 mg/kg) resulted in a CAT activity of 13.98 nmol/min/mL, which was not significantly different from the ACR group, indicating a minimal protective effect. In contrast, the higher dose (360 mg/kg) restored CAT activity to 21.64 nmol/min/mL, a significant increase compared with the ACR group, recovering approximately 52% of normal activity. Overall, the BSFL extract showed no adverse effects, as CAT activity in the group receiving only the high-dose extract remained comparable to the normal control. This finding indicates that the extract does not alter the basal antioxidant enzyme balance and lacks intrinsic toxicity. Collectively, these results demonstrate that the n-hexane oil extract of BSFL has a significant, dose-dependent protective effect against ACR-induced oxidative stress by preserving hepatic catalase activity.

**Figure 2.**
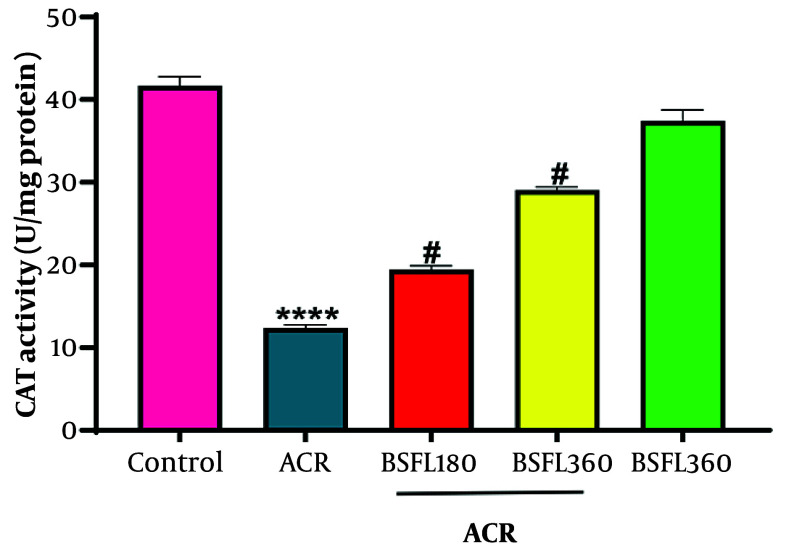
The effect of the n-hexane oil extract of Black Soldier Fly Larvae (BSFL) at two different concentrations (180 and 360 mg/kg) on hepatic catalase activity in rats exposed to ACR. Values are expressed as mean ± SD; n = 7. *Significant difference in comparison with the control group (**** P < 0.0001). #Significant difference in comparison with the ACR group (# P < 0.05).

#### 4.2.2. Reduced *Glutathione* Activity Assay

As shown in Figure 3, ACR administration induced profound oxidative stress, characterized by severe depletion of hepatic GSH. The GSH level in the ACR group plummeted to 0.312 µmol/mg protein, representing an 88.9% decrease compared with the normal control group (2.80 µmol/mg protein). Co-treatment with the BSFL extract effectively counteracted this depletion in a dose-dependent manner. The lower dose (180 mg/kg) significantly restored GSH levels to 0.705 µmol/mg protein, a 126% increase over the ACR group. The higher dose (360 mg/kg) produced a markedly stronger effect, elevating GSH to 1.652 µmol/mg protein—a 429% increase compared with the ACR group—restoring levels to nearly 59% of normal. Notably, treatment with the high-dose extract alone did not affect basal GSH levels (2.72 µmol/mg protein), confirming that the extract does not provoke oxidative stress or disrupt the endogenous antioxidant system. Thus, these findings demonstrate that the n-hexane oil extract of BSFL confers significant hepatoprotection against ACR-induced oxidative stress by preserving and restoring glutathione reserves. This potent, dose-dependent activity suggests that the extract may act by providing antioxidant constituents, supporting GSH synthesis, or enhancing the glutathione redox cycle. The absence of intrinsic toxicity further supports its therapeutic potential.

**Figure 3.**
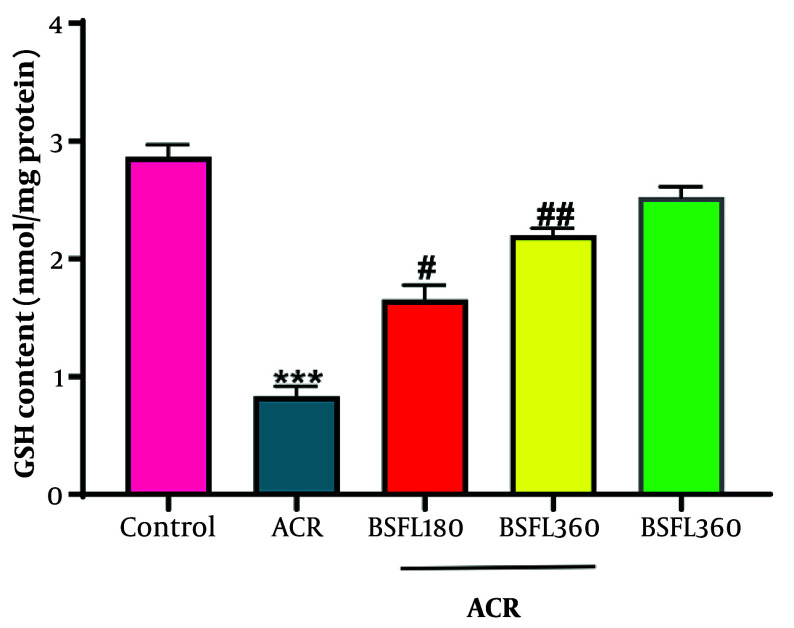
The effect of the n-hexane oil extract of Black Soldier Fly Larvae (BSFL) at two different concentrations (180 and 360 mg/kg) on hepatic GSH activity in rats exposed to ACR. Values are expressed as mean ± SD; n = 7. *Significant difference in comparison with the control group (*** P < 0.001). #Significant difference in comparison with the ACR group (# P < 0.05; ## P < 0.01).

#### 4.2.3. *Glutathione Peroxidase* Activity Assay

*Glutathione peroxidase*, a critical enzyme that utilizes glutathione to neutralize hydrogen peroxide and lipid peroxides, was severely inhibited by ACR intoxication. Co-administration of the BSFL extract significantly protected and restored GPx activity in a dose-dependent manner. The extract alone demonstrated compatibility with the normal hepatic enzymatic environment. The results provide strong evidence that ACR-induced hepatotoxicity involves direct impairment of the glutathione antioxidant system, specifically through inhibition of *Glutathione Peroxidase*. The n-hexane oil extract of BSFL larvae provided significant, dose-dependent protection against this suppression. By preserving GPx activity, the extract helps maintain the liver's ability to detoxify harmful peroxides, thereby preventing further oxidative damage to lipids, proteins, and DNA. This protective effect on GPx, together with the previously observed restoration of glutathione (GSH) levels, indicates that the extract acts comprehensively to support and enhance the glutathione redox cycle. This mechanism is a key factor in its overall hepatoprotective efficacy against ACR-induced oxidative stress (Figure 4).

**Figure 4.**
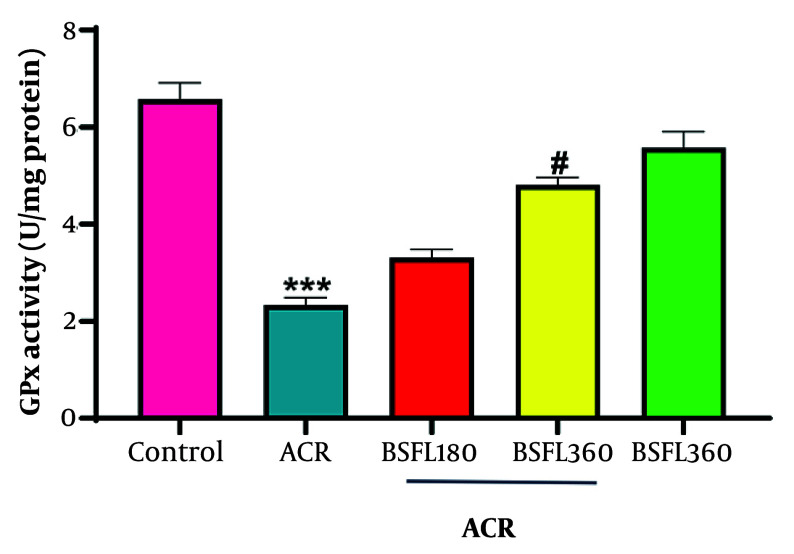
The effect of the n-hexane oil extract of Black Soldier Fly Larvae (BSFL) at two different concentrations (180 and 360 mg/kg) on hepatic GPx activity in rats exposed to ACR. Values are expressed as mean ± SD; n = 7. *Significant difference in comparison with the control group (*** P < 0.001). #Significant difference in comparison with the ACR group (# P < 0.05).

#### 4.2.4. *Superoxide Dismutase* Activity Assay

The results demonstrate that ACR-induced hepatotoxicity involves a severe compromise of the enzymatic antioxidant defense system, specifically through inhibition of *Superoxide Dismutase*. ACR intoxication resulted in a marked reduction in SOD activity. Co-administration of the BSFL extract significantly preserved SOD activity in a clear, dose-dependent manner. By preserving SOD activity, the extract helps maintain the critical balance of neutralizing superoxide radicals, thereby reducing subsequent oxidative chain reactions. The extract alone had no adverse effect on normal enzyme function. This protective effect on a key antioxidant enzyme, together with the extract's safety profile, supports its role as a potent hepatoprotective agent against ACR-induced oxidative stress (Figure 5).

**Figure 5.**
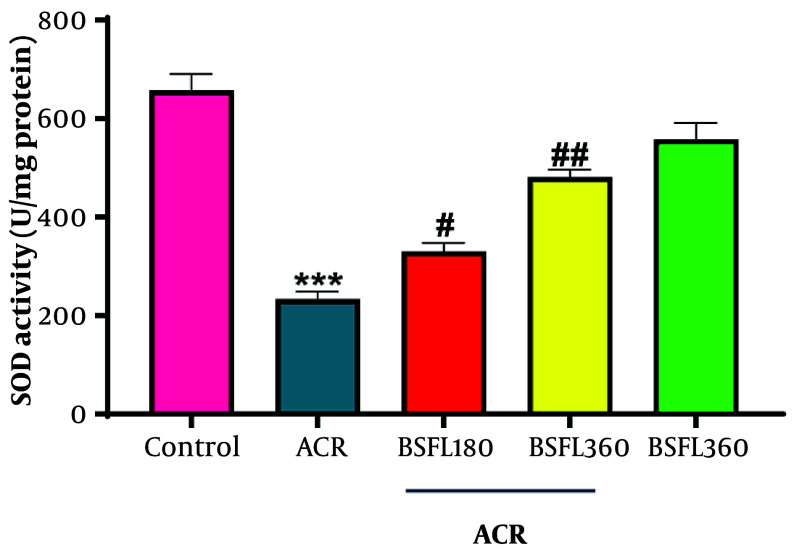
The effect of the n-hexane oil extract of Black Soldier Fly Larvae (BSFL) at two different concentrations (180 and 360 mg/kg) on hepatic SOD activity in rats exposed to ACR. Values are expressed as mean ± SD; n = 7. *Significant difference in comparison with the control group (*** P < 0.001). #Significant difference in comparison with the ACR group (# P < 0.05; ## P < 0.01).

### 4.3. Lipid Peroxidation

#### 4.3.1. Malondialdehyde Activity Assay

The results provide evidence that ACR-induced hepatotoxicity is mediated by a significant increase in oxidative stress and lipid peroxidation. The n-hexane oil extract of BSFL larvae conferred a strong, dose-dependent protective effect against this damage. The significant reduction in MDA levels with co-treatment demonstrates the extract's antioxidant capacity, stabilizing cell membranes and preventing lipid peroxidation. The ability of the extract alone to lower MDA below control levels suggests that it has intrinsic antioxidant activity. These findings support the role of the BSFL extract in ameliorating ACR-induced oxidative hepatic damage (Figure 6).

**Figure 6.**
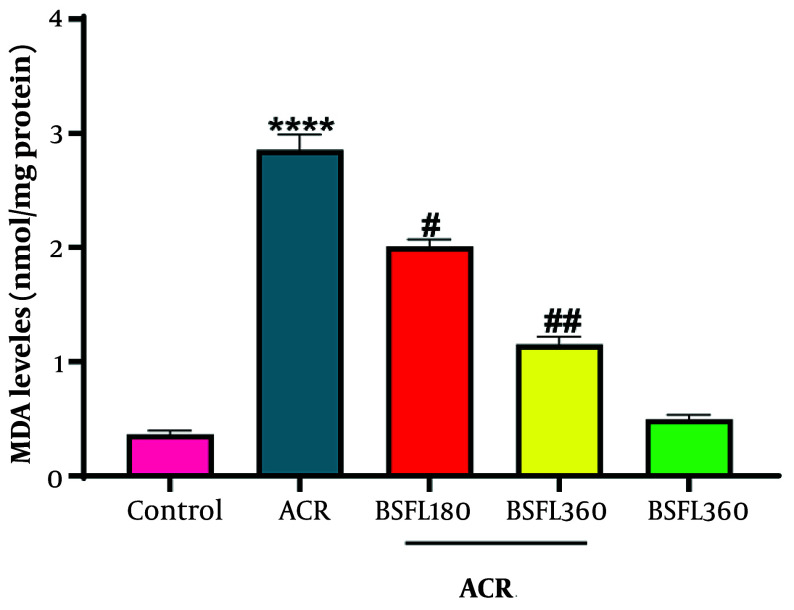
The effect of the n-hexane oil extract of Black Soldier Fly Larvae (BSFL) at two different concentrations (180 and 360 mg/kg) on hepatic MDA activity in rats exposed to ACR. Values are expressed as mean ± SD; n = 7. *Significant difference in comparison with the control group (**** P < 0.0001). #Significant difference in comparison with the ACR group (# P < 0.05; ## P < 0.01).

ACR intoxication resulted in a severe increase in lipid peroxidation, as evidenced by a marked rise in MDA levels. Administration of ACR caused a massive and significant increase in hepatic MDA levels to 2.000 µmol/mg protein, compared with the normal control group (0.70 µmol/mg protein). This represents a ~186% increase, confirming that ACR induces severe oxidative stress, leading to extensive damage to cell membranes through lipid peroxidation.

Co-treatment with the lower dose of BSFL extract (180 mg/kg) significantly reduced ACR-induced lipid peroxidation compared with the ACR group, representing a 22% reduction. Co-treatment with the higher dose (360 mg/kg) markedly reduced MDA levels to 1.114 µmol/mg protein, a 44.3% reduction compared with the ACR-only group, bringing MDA levels closest to normality. The extract alone showed no pro-oxidant effect, exhibiting an MDA level of 0.358 µmol/mg protein, which is lower than the control group, confirming its safety and intrinsic antioxidant properties.

#### 4.3.2. Nitric Oxide Level

Nitric oxide, a key signaling molecule involved in inflammation and vasodilation, was significantly altered by the treatments. ACR intoxication resulted in a significant increase in NO levels (P < 0.001), consistent with previous reports that acrylamide upregulates inducible nitric oxide synthase (iNOS) expression. Co-administration of the BSFL extract significantly attenuated this elevation in a dose-dependent manner, bringing NO levels toward normal values (Figure 7).

**Figure 7.**
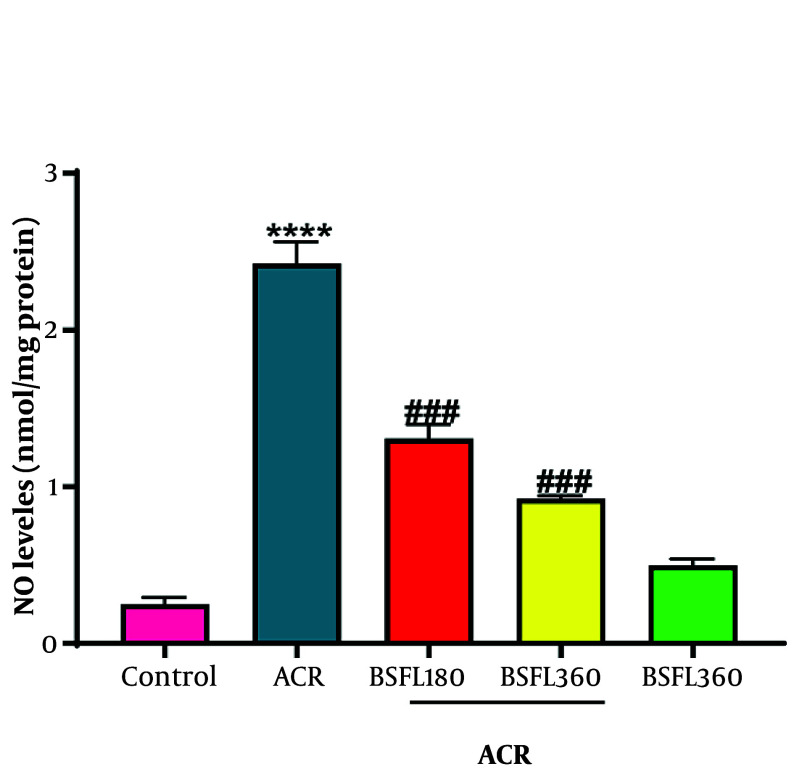
The effect of the n-hexane oil extract of Black Soldier Fly Larvae (BSFL) at two different concentrations (180 and 360 mg/kg) on hepatic NO level in rats exposed to ACR. Values are expressed as mean ± SD; n = 7. *Significant difference in comparison with the control group (**** P < 0.0001). #Significant difference in comparison with the ACR group (### P < 0.001).

The results reveal a complex modulatory effect of the BSFL extract on nitric oxide metabolism. Normalization of NO levels may contribute to preservation of liver function, as nitric oxide is important for maintaining hepatic blood flow and cell signaling.

The extremely high level of NO in the group receiving the extract alone suggests that the BSFL extract contains potent compounds that stimulate nitric oxide synthesis. Although excessive NO can contribute to nitrosative stress, the absence of toxicity (as confirmed by other markers and histopathological assessment) suggests a controlled and potentially beneficial upregulation.

Overall, the n-hexane oil extract of BSFL larvae shows a strong, dose-dependent ability to modulate nitric oxide levels. It attenuates the ACR-induced elevation of NO and, when administered alone, acts as a potent inducer of NO synthesis. This property likely contributes to its hepatoprotective effect by improving hepatic perfusion and restoring crucial cell signaling pathways disrupted by ACR toxicity. The ability to substantially elevate NO without causing apparent harm highlights a novel and important pharmacological property of this extract.

### 4.4. Inflammatory Markers (Liver Homogenate)

#### 4.4.1. Effect of BSFL Extract on Hepatic IL-1β Level

The inflammatory response in liver tissue was assessed by measuring the levels of the pro-inflammatory cytokine IL-1β (6). The results are summarized in Figure 8. ACR administration induced a severe inflammatory response, as evidenced by a significant increase (P < 0.001) in hepatic IL-1β levels (53.31 and 42.88 pg/mg protein) compared with the control group (12.54 and 14.71 pg/mg protein). Co-treatment with the n-hexane oil extract of black soldier fly larvae at a dose of 180 mg/kg (ACR 180) resulted in a notable attenuation of this increase. IL-1β levels in the ACR 180 group (36.94 and 32.84 pg/mg protein) were lower than those in the ACR-only group, indicating reduced inflammation. This protective effect was more pronounced and statistically significant (P < 0.01) at the higher dose of 360 mg/kg (ACR 360). IL-1β levels in the ACR 360 group (29.82 and 19.58 pg/mg protein) were markedly reduced, approaching those observed in the healthy control group. These findings demonstrate a clear, dose-dependent ameliorative effect of the extract on ACR-induced hepatic inflammation.

**Figure 8.**
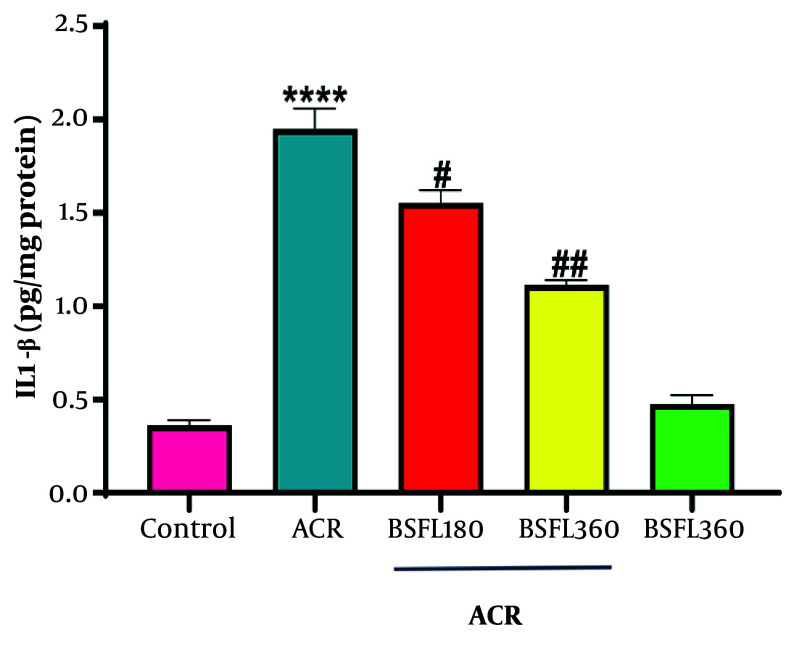
The effect of the n-hexane oil extract of Black Soldier Fly Larvae (BSFL) at two different concentrations (180 and 360 mg/kg) on hepatic IL-1β level in rats exposed to ACR. Values are expressed as mean ± SD; n = 7. *Significant difference in comparison with the control group (**** P < 0.0001). #Significant difference in comparison with the ACR group (# P < 0.05; ## P < 0.01).

#### 4.4.2. Effect of BSFL Extract on Hepatic IL-6 Level

Hepatic levels of the pro-inflammatory cytokine Interleukin-6 (IL-6) were measured to further evaluate the inflammatory status (Figure 9). Administration of ACR resulted in a dramatic and significant increase (P < 0.001) in IL-6 concentrations (37.82 and 41.57 pg/mg protein) compared with the control group (3.32 and 7.11 pg/mg protein), confirming severe hepatic inflammation. Treatment with the n-hexane oil extract of black soldier fly larvae at a dose of 180 mg/kg (ACR 180) significantly mitigated (P < 0.01) this elevation, reducing IL-6 levels to 25.69 and 23.13 pg/mg protein. Notably, the higher dose of 360 mg/kg (ACR 360) exhibited a more potent protective effect, decreasing IL-6 levels to 12.41 and 14.68 pg/mg protein. This reduction was highly significant (P < 0.001) compared with the ACR group and demonstrates a clear, dose-dependent anti-inflammatory action of the extract.

**Figure 9.**
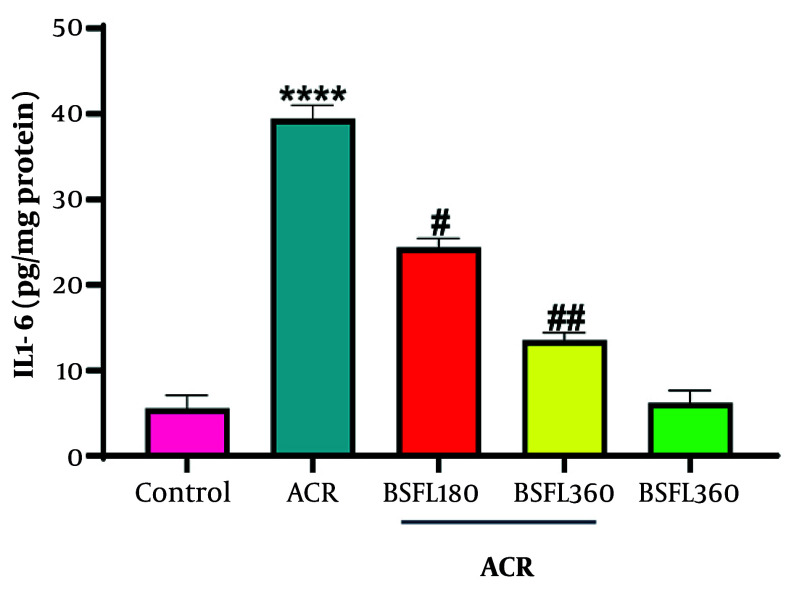
The effect of the n-hexane oil extract of Black Soldier Fly Larvae (BSFL) at two different concentrations (180 and 360 mg/kg) on hepatic IL-6 level in rats exposed to ACR. Values are expressed as mean ± SD; n = 7. *Significant difference in comparison with the control group (**** P < 0.0001). #Significant difference in comparison with the ACR group (# P < 0.05; ## P < 0.01).

#### 4.4.3. Effect of BSFL Extract on Hepatic TNF-Α Levels

The impact on hepatic inflammation was further assessed by measuring the levels of the key pro-inflammatory cytokine TNF-α. The results are presented in Figure 10. Induction of hepatotoxicity with ACR led to a profound increase in TNF-α concentrations (39.41 and 67.58 pg/mg protein) compared with the control group (8.96 and 12.63 pg/mg protein) (P > 0.05). Co-administration of the n-hexane oil extract of black soldier fly larvae at a dose of 180 mg/kg (ACR 180) attenuated this increase, with TNF-α levels of 27.58 and 37.10 pg/mg protein. The protective effect was more pronounced at the higher dose of 360 mg/kg (ACR 360). Treatment with this dose reduced TNF-α levels to 23.05 pg/mg protein, demonstrating a clear and potent dose-dependent anti-inflammatory effect.

**Figure 10.**
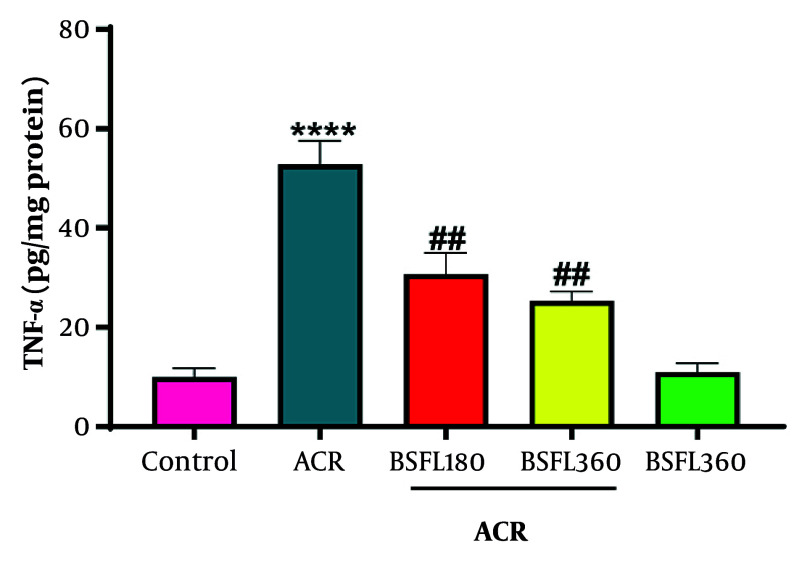
The effect of the n-hexane oil extract of Black Soldier Fly Larvae (BSFL) at two different concentrations (180 and 360 mg/kg) on hepatic TNF-α level in rats exposed to ACR. Values are expressed as mean ± SD; n = 7. *Significant difference in comparison with the control group (**** P < 0.0001). #Significant difference in comparison with the ACR group (## P < 0.01).

### 4.5. Western Blot Analysis of Endoplasmic Reticulum Stress-Related Molecular Markers

Western blot analysis was performed to evaluate the expression of key signaling proteins involved in ER stress, apoptosis, and inflammation. The results are presented in Figure 11. The ACR-intoxicated group exhibited pronounced upregulation of pro-apoptotic and pro-inflammatory signaling molecules compared with the normal control group. Specifically:

**Figure 11.**
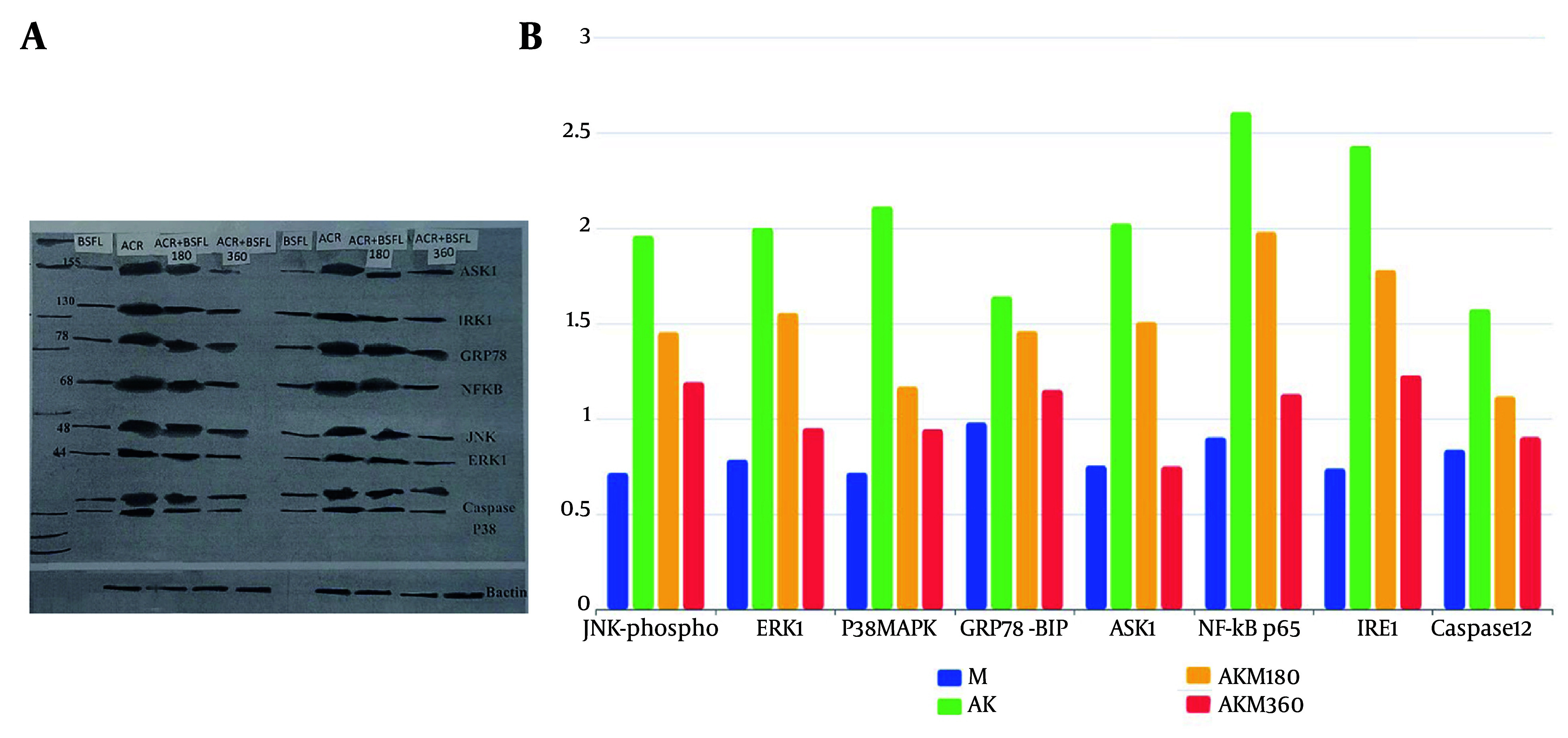
A, Representative western blot bands for ASK1, phospho-JNK, p38 MAPK, GRP78/BiP, NF-κB p65, Caspase 12, ERK1, and β-actin (loading control) in liver tissue of experimental groups. B, Quantitative densitometric analysis of protein expression normalized to β-actin. Data are presented as mean ± SD. (M: control; ACR: acrylamide alone; ACR180: ACR + BSFL 180 mg/kg; ACR360: ACR + BSFL 360 mg/kg).

- The expression of phospho-JNK and ASK1 increased approximately 2.5-fold

- The ER stress marker GRP78/BiP was upregulated about 2-fold

- The inflammatory transcription factor NF-κB p65 showed a 1.8-fold increase

- The apoptotic executioner Caspase 12 was elevated by approximately 1.7-fold.

In contrast, expression of ERK1, a kinase associated with cell survival and proliferation, was downregulated in the ACR group, shifting the balance toward cell death. Treatment with the BSFL extract, particularly at the higher dose (360 mg/kg), dose-dependently attenuated the ACR-induced upregulation of these proteins. Expression of ASK1, GRP78, NF-κB p65, phospho-JNK, p38 MAPK, and Caspase 12 was significantly reduced in the ACR + BSFL groups compared with the ACR group. Furthermore, the BSFL extract helped restore pro-survival ERK1 levels toward normal values. These findings suggest that the protective effect of the BSFL extract is associated with changes in critical signaling pathways. Suppression of ASK1 and GRP78 indicates alleviation of ER stress; downregulation of NF-κB p65 signifies anti-inflammatory activity; and inhibition of the JNK and p38 pathways, together with reduced Caspase 12 expression, suggests an anti-apoptotic effect. It is important to note that changes in protein expression levels indicate potential involvement of these signaling molecules but do not directly demonstrate pathway activation or inhibition. Causal interpretation would require additional experiments using specific pathway inhibitors or genetic approaches.

### 4.6. Histopathological Examination

#### 4.6.1. Masson Trichrome Staining

Control liver specimens maintained a normal structural appearance, with only negligible collagen accumulation near vascular regions, confirming the absence of fibrosis. In contrast, the ACR group exhibited a severe and widespread increase in collagen fibers, heavily concentrated in the portal tracts, indicative of substantial fibrotic damage (Figure 12A). Animals treated solely with BSFL 360 showed liver histology that was almost identical to that of healthy controls, with minimal collagen, highlighting the extract's baseline characteristics. In the diseased model, both the lower (180 mg/kg) and higher (360 mg/kg) doses of BSFL extract clearly mitigated collagen accumulation compared with the untreated ACR group. The therapeutic effect was particularly evident at 360 mg/kg, which produced a marked decline in fibrotic markers, approaching the healthy state of the control livers (Figure 12B-C).

**Figure 12.**
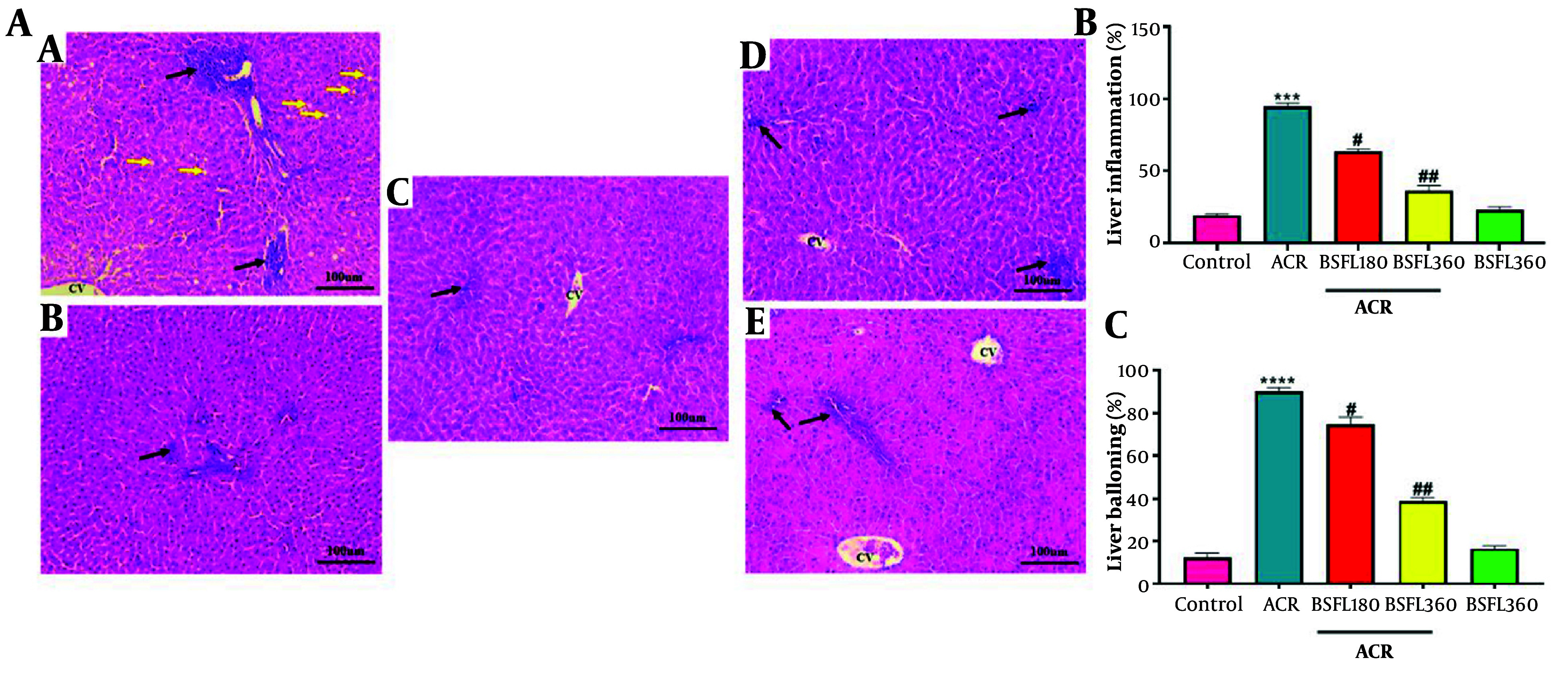
Histological findings of liver tissues after H&E staining (magnification ×100) in different experimental groups. AA, ACR-induced model showing ballooning degeneration (yellow arrows, empty spaces indicating fat accumulation and cell enlargement) and inflammatory cell accumulation around vessels (black arrows). AB, BSFL 360 alone showing lower inflammatory cells. AC, Control showing normal liver architecture. AD, ACR + BSFL 180 showing reduced inflammatory cells. AE, ACR + BSFL 360 showing further reduced inflammatory cells. B, Quantitative analysis of liver inflammation (%). C, Quantitative analysis of ballooning degeneration (%). Data are expressed as mean ± SEM (n = 6). ***P < 0.001 compared to the control group; # P < 0.05 and ## P < 0.01 compared to the ACR group (one-way ANOVA followed by Tukey's post-hoc test).

#### 4.6.2. H&E Staining

In the control group, hepatocytes appeared healthy and well organized, with intact architecture, clearly defined central veins, and a complete absence of inflammatory cell activity. This normal appearance contrasted sharply with the ACR group, in which liver sections showed clear signs of injury, including widespread hepatocellular degeneration, disruption of orderly tissue structure, and moderate to severe inflammatory cell infiltration. Animals that received only BSFL 360 largely maintained preserved hepatic tissue, exhibiting near-normal cellular morphology with only negligible signs of inflammation. Most notably, in the groups that received both the ACR challenge and subsequent BSFL treatment, a clear therapeutic effect was observed; both the 180 and 360 mg/kg doses produced visible improvement in liver microarchitecture, with reduced cellular degeneration and substantial attenuation of the inflammatory response, an effect that was markedly more pronounced at the higher 360 mg/kg dose (Figure 13).

**Figure 13.**
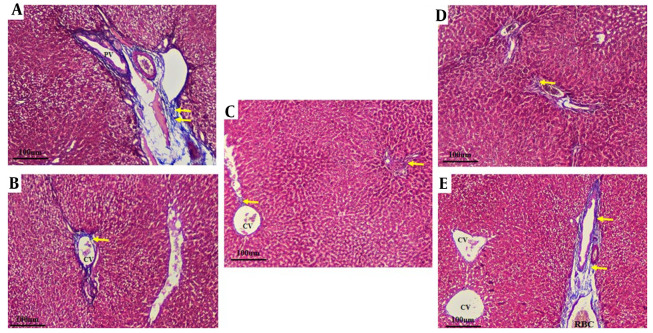
Histological findings of liver tissues after Masson's trichrome staining (magnification ×100) in different experimental groups. A, ACR-induced model showing extensive collagen deposition (yellow arrows). B, BSFL 360 alone showing minimal collagen. C, Control showing normal liver architecture with no fibrosis. D, ACR + BSFL 180 showing reduced collagen deposition. E, ACR + BSFL 360 showing marked reduction in collagen deposition. CV: central vein; PV: portal vein. Yellow arrows indicate collagen fibers.

## 5. Discussion

Frying foods generates ACR, a neurotoxic compound that can also damage the gut and potentially the liver as part of its systemic toxicity. Globally, the Black Soldier Fly Larva (BSFL) is acknowledged as a promising insect protein source used to recycle food waste, which often contains ACR from fried products (18, 19). ACR is a harmful chemical compound that can cause liver toxicity via various mechanisms, such as oxidative stress and programmed cell death (20). The larval oil-based extract of this insect is a rich source of fatty acids such as lauric acid, palmitic acid, and linoleic acid. Furthermore, the conversion of lauric acid in larval oil to monolaurin can induce the activity of antioxidant enzymes and reduce inflammatory factors (17).

The present study provides evidence that the n-hexane oil extract of Black Soldier Fly (BSF) larvae confers significant hepatoprotection against ACR-induced toxicity in rats. The protective efficacy is demonstrated through a multifaceted approach, including restoration of antioxidant defenses, reduction of oxidative stress and lipid peroxidation, suppression of pro-inflammatory cytokine release, and association with changes in key intracellular signaling pathways involved in stress responses, inflammation, and apoptosis. Importantly, this foundational study intentionally used the total n-hexane extract of BSFL rather than an isolated single compound. This approach is justified because hepatoprotective activity against a multifaceted toxin such as ACR is likely attributable to the combined, and potentially synergistic, actions of diverse bioactive constituents. The BSFL oil used here is characterized by a unique lipid profile, notably rich in medium-chain fatty acids such as lauric acid (comprising a large proportion of its fatty acid content) and contains other lipophilic bioactive compounds, including tocopherols (9, 10). The broad-spectrum, dose-dependent protection observed across oxidative, inflammatory, apoptotic, and histopathological endpoints supports the concept that the total extract may exert a more comprehensive therapeutic effect than any single purified component, possibly through multi-target interactions. Although future bioassay-guided fractionation studies are warranted to identify the primary active molecule(s), the current work provides crucial proof of concept for the value of the total BSFL extract as a sustainable, multi-target natural product for mitigating chemical-induced hepatotoxicity.

The induction of hepatotoxicity by ACR is a complex process involving oxidative stress, which subsequently triggers ER stress, inflammation, and apoptosis. The observed upregulation of ASK1, a redox-sensitive kinase (18), and the efficacy of the BSFL extract in mitigating these changes may be attributed to its likely rich composition of bioactive compounds, such as antimicrobial peptides, lauric acid, and other medium-chain fatty acids, which are known for their antioxidant and anti-inflammatory properties (1). The primary impact of ACR toxicity appears to be a significant disturbance of the hepatic antioxidant system. Our findings show that ACR intoxication led to severe depletion of key enzymatic (CAT, SOD, GPx) and non-enzymatic (GSH) antioxidants. The dramatic decrease in the activities of CAT, SOD, and GPx indicates an overwhelmed defense system incapable of neutralizing the surge of reactive oxygen species (17) produced by ACR. This is further corroborated by the significant accumulation of MDA, a terminal product of lipid peroxidation, confirming extensive damage to cellular membranes. *Glutathione* (GSH) is a prevalent thiol tripeptide found in numerous cell types, such as cardiomyocytes, hepatocytes, and erythrocytes. The glutathione system plays a crucial role in regulating the levels of O_2_•^-^ and H_2_O_2_, ensuring they remain at physiological concentrations essential for tissue repair and immune defense. Consequently, the ratio of oxidized to reduced glutathione serves as an indicator of a cell's redox state and may be a useful tool for evaluating oxidative stress, as well as a potential target for drug-based antioxidant therapies. Meanwhile, Lee et al. showed that BSFL extract had neither hepatotoxic nor hepatoprotective effects on MDA levels (20). Notably, the safety profile of the BSFL extract itself is a critical finding. Administration of the high dose alone did not adversely affect any of the measured parameters; in fact, it slightly enhanced basal antioxidant status (reduced MDA below control levels) without causing hyperactivation or imbalance, confirming its non-toxic nature (21).

A particularly intriguing finding was the extract's profound modulatory effect on nitric oxide levels. ACR significantly increased NO levels, consistent with previous reports that acrylamide upregulates inducible nitric oxide synthase (iNOS) expression. Excessive NO production can contribute to nitrosative stress and tissue injury. Co-administration of BSFL extract significantly attenuated this elevation in a dose-dependent manner, bringing NO levels toward normal values. This suggests that the BSFL extract exerts its protective effect partly by preventing excessive NO overproduction, rather than by restoring NO levels. By quenching reactive oxygen species and reducing oxidative stress, these compounds may downregulate iNOS expression, thereby preventing excessive NO production. The absence of toxicity in the extract-only group confirms its safety profile (18). Our finding that the extract alone could lower basal MDA levels below those of the control group suggests that it contains intrinsic, potent antioxidant compounds, such as flavonoids, terpenoids, or unique fatty acids present in the n-hexane fraction of BSF larvae oil (22).

In addition to oxidative stress, hepatotoxicity induced by ACR is marked by a strong inflammatory response. Previous research has demonstrated that inflammatory cytokines, including IL-1β, IL-6, and TNF-α, are elevated due to ACR exposure. Our data show a marked elevation in these pro-inflammatory cytokines in the livers of ACR-intoxicated rats. This abundant secretion of cytokines contributes to secondary liver tissue damage and amplifies the initial injury. The BSFL extract demonstrated a potent, dose-dependent anti-inflammatory effect, significantly attenuating the rise of all three cytokines. This effect is likely associated with inhibition of the NF-κB signaling pathway, as supported by our Western blot analysis. Activation of NF-κB results in the induction of transcriptional targets of pro-inflammatory genes, including those for IL-1β, IL-6, and TNF-α, which play crucial roles in regulating the host immune and inflammatory responses. The ability of the BSFL extract to suppress NF-κB activation provides a molecular explanation for the observed reduction in cytokine levels.

The quantitative data provide evidence for the molecular mechanism of ACR-induced hepatotoxicity and its mitigation by the BSFL extract. The dramatic ~2.5-fold increase in ASK1 and JNK-phosphate in the ACR group underscores the critical role of oxidative stress-induced apoptotic signaling. ASK1 is a redox-sensitive kinase that activates both JNK and p38 pathways, leading to cell death. The concomitant rise in GRP78/BiP and Caspase 12 confirms the involvement of ER stress-mediated apoptosis (23). The Western blot analysis further revealed the intracellular signaling mechanisms underlying hepatoprotection. The upregulation of GRP78, a key marker of endoplasmic reticulum stress (24), and its upstream regulator ASK1 in the ACR group indicates that ER stress is a significant contributor to ACR-induced apoptosis. Hone et al. reported that inhibition of ASK1 provides liver protection during stressful conditions. In addition, elevated ASK1 expression correlates with increased expression of the ER stress marker GRP78 (25). The BSFL extract effectively suppressed this ER stress response. Furthermore, the extract was associated with attenuation of the JNK and p38 MAPK pathways, which are stress-sensitive kinases that promote apoptosis and inflammation. According to another study, inhibition of ASK1 provides hepatoprotective and anti-inflammatory effects by activating the NLRP3 signaling pathway, which leads to a reduction in liver cell death and fibrosis. Additionally, both p38 and JNK can enhance fibrogenic gene expression by phosphorylating the nuclear transcription factors ATF2 and c-Jun. Another study (26) showed that low expression of p38 and JNK genes and proteins demonstrates a protective effect against hepatic ischemia damage. Concurrently, our extract was associated with changes in the levels of ERK1, a kinase generally associated with cell survival and proliferation. The shift in balance from pro-apoptotic signaling pathways (JNK, p38) to pro-survival pathways (ERK) is a vital mechanism through which the BSFL extract maintains hepatocyte viability.

The current study demonstrates that the n-hexane oil extract of Black Soldier Fly Larvae confers notable hepatoprotective effects against acrylamide-induced oxidative stress, inflammation, and histopathological disruptions in rat liver, as evidenced by marked improvement in both H&E-stained (Figure 12) and Masson's trichrome-stained (Figure 13) sections. Collagen accumulation (fibrosis) and inflammatory infiltration, hallmarks of acrylamide hepatotoxicity, were markedly attenuated by BSFL administration, especially at the higher dose (27).

These findings are consistent with previous reports on the antioxidant and anti-inflammatory properties of insect-derived n-hexane oil mixtures and phytochemicals such as quercetin, thymoquinone, and curcumin, which have shown similar protective effects against acrylamide and other hepatotoxins. Histological parallels can be drawn with studies in which natural compounds reduced collagen deposition and preserved hepatic morphology in toxin-induced models. In particular, the dose-dependent effect, whereby higher concentrations of the n-hexane extract better ameliorated ACR-induced damage, aligns with observations in plant extract studies such as the n-hexane fraction of *Morus nigra* and Alstonia boonei (17, 28).

Mechanistically, the BSFL oil extract likely exerts its effects via mitigation of ROS production, stabilization of mitochondrial membranes, and downregulation of fibrogenic and inflammatory signaling pathways, as supported by both histological preservation and biochemical evidence from similar studies (27, 28).

Finally, Masson's trichrome and H&E histopathology confirm that the n-hexane extract of BSFL larvae significantly protects liver tissue against ACR-induced fibrosis and inflammation. These results support further investigation into the molecular pathways underlying this protection and suggest potential translational applications for BSFL extracts as novel therapeutic agents against chemically induced hepatotoxicity.

In conclusion, the n-hexane oil extract of BSF larvae exerts a robust protective effect against ACR-induced hepatotoxicity through a synergistic combination of mechanisms. It functions as a potent antioxidant, directly and indirectly bolstering the cellular defense system against ROS. It acts as an effective anti-inflammatory agent, likely associated with inhibition of the NF-κB pathway. Moreover, it is associated with changes in critical cell signaling pathways, alleviating ER stress and shifting the balance from apoptosis toward cell survival. The consistent dose-dependent efficacy and the absence of intrinsic toxicity highlight the therapeutic potential of BSFL extract as a natural protective agent against chemical-induced liver injury.

The present study has some limitations. First, we did not perform independent chemical characterization (fatty acid profiling, quantification of lauric acid, residual solvent analysis) of the BSFL n-hexane extract used. Therefore, the estimated lauric acid content is based on published literature rather than direct measurement. Second, residual n-hexane was not quantified, although the extract was thoroughly dried under reduced pressure before administration. Third, this is a preliminary proof-of-concept study with a limited sample size (n = 7 per group). Additionally, complete blinding was not achieved for treatment administration and histopathological assessment, which may introduce detection bias, although biochemical and western blot analyses were performed blinded. Despite these limitations, the consistent and dose-dependent protective effects observed across multiple independent endpoints provide strong evidence for the hepatoprotective efficacy of the BSFL extract.

Furthermore, a crude extract was used rather than purified compounds, and no pathway inhibitors, gene knockdown, or rescue experiments were performed. Therefore, the observed changes in protein expression represent associations rather than direct causal evidence of pathway modulation. Future studies using targeted inhibitors or genetic approaches (e.g., siRNA for GRP78 or ASK1) are needed to confirm the specific signaling pathways involved. Future studies should also focus on identifying the specific bioactive compounds responsible for these effects and elucidating their precise molecular targets, particularly their interaction with the Nrf2 and NF-κB pathways. Bioassay-guided fractionation is essential to pinpoint the precise molecule(s) responsible for hepatoprotection, building directly on the foundational evidence provided here.
